# Regional differences in acute corticosterone-induced dendritic remodeling in the rat brain and their behavioral consequences

**DOI:** 10.1186/1471-2202-15-65

**Published:** 2014-05-22

**Authors:** Hyejin Kim, Jee Hyun Yi, Kyuhyun Choi, Seokheon Hong, Ki Soon Shin, Shin Jung Kang

**Affiliations:** 1 Department of Biology, Department of Life and Nanopharmaceutical Sciences, Kyung Hee University, 130-701 Seoul, Republic of Korea; 2 Department of Molecular Biology, Sejong University, 143-747 Seoul, Republic of Korea

**Keywords:** Corticosterone, Basolateral amygdala, Medial prefrontal cortex, Stress, Working memory, Anxiety

## Abstract

**Background:**

Glucocorticoid released by stressful stimuli elicits various stress responses. Acute treatment with a single dose of corticosterone (CORT; predominant glucocorticoid of rats) alone has previously been shown to trigger anxiety behavior and robust dendritic hypertrophy of neurons in the basolateral amygdala (BLA). Neurons in the medial prefrontal cortex (mPFC) are also known to be highly sensitive to stress and regulate anxiety-like behaviors. Nevertheless, we know less about acute CORT-induced structural changes of other brain regions and their behavioral outcomes. In addition, the temporal profile of acute CORT effects remains to be examined. The current study investigates time course changes of dendritic architectures in the stress vulnerable brain areas, the BLA and mPFC, and their behavioral consequences after acute treatment with a single dose of CORT.

**Results:**

Acute CORT treatment produced delayed onset of dendritic remodeling in the opposite direction in the BLA and mPFC with different time courses. Acute CORT induced dendritic hypertrophy of BLA spiny neurons, which was paralleled by heightened anxiety, both peaked 12 days after the treatment. Meanwhile, CORT-induced dendritic atrophy of mPFC pyramidal neurons peaked on day 6, concomitantly with impaired working memory. Both changed dendritic morphologies and altered behavioral outcomes were fully recovered.

**Conclusion:**

Our results suggest that stress-induced heightened anxiety appears to be a functional consequence of dendritic remodeling of BLA neurons but not that of mPFC. Instead, stress-induced dendritic atrophy of mPFC neurons is relevant to working memory deficit. Therefore, structural changes in the BLA and the mPFC might be specifically associated with distinct behavioral symptoms observed in stress-related mental disorders. Remarkably, stress-induced dendritic remodeling in the BLA as well as mPFC is readily reversible. The related behavioral outcomes also follow the similar time course in a reversible manner. Therefore, further studies on the cellular mechanism for the plasticity of dendrites architecture might provide new insight into the etiological factors for stress-related mental illness such as posttraumatic stress disorder (PTSD).

## Background

Stress results in the activation of hypothalamus-pituitary-adrenal axis, leading to secretion of a stress hormone, glucocorticoid [1,2]. Glucocorticoids bind to glucocorticoid receptors, inducing various stress responses. Chronic glucocorticoid treatment causes dendritic atrophy in the hippocampus and spatial memory deficits observed in stressed animals, which suggests that glucocorticoid is critical in stress-induced hippocampal damage [3,4]. While earlier studies on the effects of stress and glucocorticoid on the brain were largely focused on the hippocampus, more recently other brain regions have been investigated in terms of stress-related mental illness such as PTSD [5-8].

The mPFC is involved in the integration of cognitive and emotional information for attentional processing [9,10]. Abnormal activity in the mPFC generally appears in the stress-related mental illness [11]. For instance, chronic restraint stress in rodents decreases dendritic length and number of branch points of mPFC pyramidal neurons [12]. The atrophy of mPFC is considered to be related with some behavioral alterations in PTSD patients [13,14]. Glucocorticoids play an important role in the regulation of the stress response through direct action at receptors in the mPFC [15] and activation of these receptors with chronic injections of CORT produces dendritic atrophy in mPFC neurons [16], mimicking the effect of chronic stress.

Meanwhile, chronic immobilization stress results in increased dendritic length and number of branch points of BLA spiny neurons in rodents [17,18]. In addition to the increased arborization of BLA neurons, chronic immobilization stress facilitate anxiety-like behavior [17-19]. Interestingly, chronic unpredictable stress fails to produce both dendritic elongation [18] and anxiety-like behavior [19]. These findings suggest that certain forms of stress may affect BLA hypertrophy and thereby lead to enhanced anxiety.

Even a single acute immobilization stress causes increased spine density and heightened anxiety, without changing dendritic arborization [17]. Interestingly, a previous study reported that acute treatment with a single dose of glucocorticoid elicits neuronal hypertrophy in the BLA and enhanced anxiety 12 days after the treatment [20]. This study also showed that the hypertrophic effect of acute CORT is sensitive enough to be saturated by a single dose of CORT because chronic CORT treatment for 10 days caused no further dendritic changes. Thus, dendritic remodeling of BLA neurons seems to require an incubation period after the stressful experience before they become evident and this structural change coincides with delayed anxiety behavior. In this regard, the effect of acute CORT treatment appears to be reminiscent of PTSD, in which a single traumatic event triggers changes in behaviors including anxiety that are both delayed and prolonged [21,22] and accompanied by BLA hyperactivity [23].

When stress-induced anxiety is prolonged or exaggerated, it can produce mental illness. In humans, exposure to severe stress like a life-threatening event is the precipitating factor for PTSD. However, most of people experience at least one traumatic event in their lifetime [24], yet only about 15% of those develop PTSD [25,26]. Upon exposure to traumatic stress, PTSD-like symptoms are apparent in almost all people in the days and weeks but the symptoms gradually disappear thereafter in the majority [27]. Thus, PTSD can be best described as a condition in which the process of recovery from trauma is impeded.

Although acute CORT-treatment rodent model has revealed delayed yet robust effects on both anxiety level and dendritic hypertrophy of BLA neurons, detailed temporal profiles following the acute CORT treatment have not been examined yet. Furthermore, how acute CORT treatment affects other stress-vulnerable brain regions is awaiting investigations. Therefore, in the present study, we explored the time-course effect of acute CORT treatment on dendritic architectures in the mPFC and the BLA. We also investigated the relationship between dendritic remodeling of the different brain regions and the behavioral outcomes across time.

## Results

**Figure 1:**
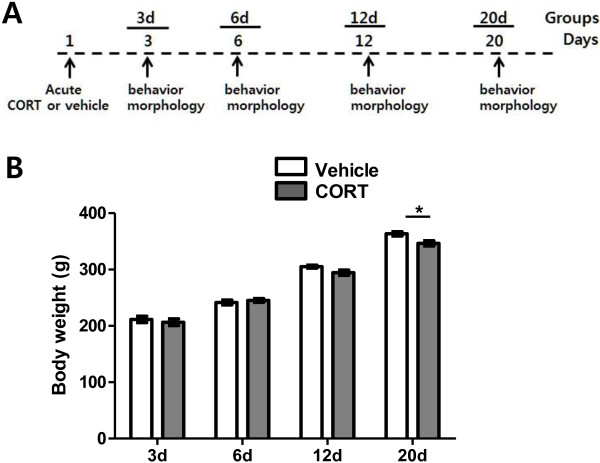
**Experimental schedule and time course change of body weight. (A)** Experimental schedule to study temporal aspects of behavioral and neuronal morphological changes after acute CORT treatment. Vehicle or CORT was subcutaneously administered on day 1 and time-course changes of behavior and dendritic arborization were measured on days 3, 6, 12 and 20. **(B)** Time course changes of body weight upon acute treatment of vehicle or CORT. Significance of difference in body weight was analyzed by using a two-way repeated-measures ANOVA. Delayed significant difference by acute CORT treatment was revealed on day 20. *, P < 0.05 (*post hoc* Bonferroni test).

Temporal aspects of behavioral and dendritic changes following acute CORT treatment have not been examined yet. Therefore, in the present study, we explored the temporal effects of acute CORT or vehicle administration by measuring dendritic architecture and behavior with variable delays, i.e., 3, 6, 12, 20 days after the treatment (Figure 1A). When we first measured a temporal change of body weight following acute CORT treatment (10 mg/kg of body weight), a two-way repeated-measures ANOVA revealed significant main effect of time (F_(3, 54)_ = 391.9, P < 0.0001) and of treatment (F_(1, 54)_ = 5.54, P < 0.05) but not of interaction (F_(3, 54)_ = 1.79, P > 0.15) (Figure 1B). Delayed significant difference in body weight by acute CORT treatment was revealed on day 20 (P < 0.05, *post hoc* Bonferroni test).

### Dendritic architecture of BLA neurons

**Figure 2:**
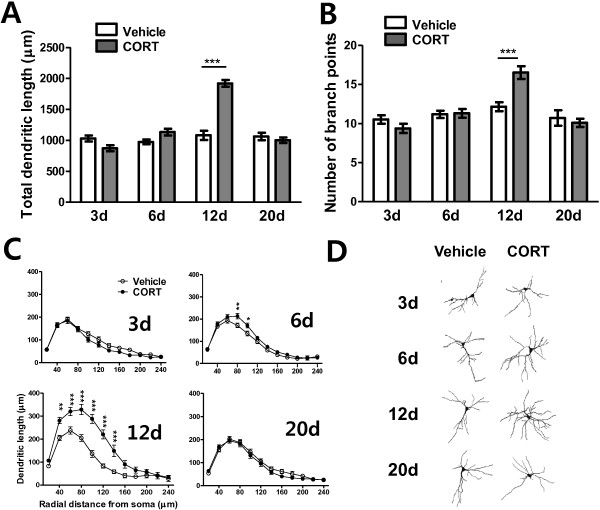
**Temporal changes in dendritic arborization of BLA neurons following acute CORT treatment.** Acute CORT treatment elicited an increase in dendritic arborization 12 days after the treatment. The increase in dendritic arborization was manifested as increases in total dendritic length **(A)** and total number of branch points **(B)**, and segmental analysis revealed an increase in dendritic arborization along a wide range of dendritic segments **(C)**. Representative camera lucida drawings of Golgi-impregnated BLA pyramidal neurons for vehicle- or CORT- treated groups with various delay periods after the treatment **(D)**.*, P < 0.05; **, P < 0.01; ***, P < 0.001 (*post hoc* Bonferroni test).

Total dendritic length (Figure 2A) and number of branch points (Figure 2B) of BLA neurons were measured at various time points following a treatment of vehicle or CORT. Seven animals were assigned to each group and 20 ~ 26 neurons (about 3 neurons per an animal) were used to measure dendritic architectures. Total dendritic length and number of branch point for each animal were averaged, from which the group averages were calculated.

Overall, hypertrophic alteration of dendritic arborization of BLA neurons upon acute CORT treatment was significantly delayed and reversible. In terms of dendritic length of BLA neurons, a two-way ANOVA revealed significant main effects of time (F_(3, 48)_ = 40.98, P < 0.0001), of treatment (F_(1, 48)_ = 25.39, P < 0.0001), and of their interaction (F_(3, 48)_ = 33.55, P < 0.0001). Number of branch points of BLA neurons also showed significant main effects of time (F_(3, 48)_ = 16.43, P < 0.0001), and of their interaction (F_(3, 48)_ = 7.39, P = 0.0004), though main effect of treatment did not reach statistical significance (F_(1, 48)_ = 2.167, P > 0.1).

**Figure 3:**
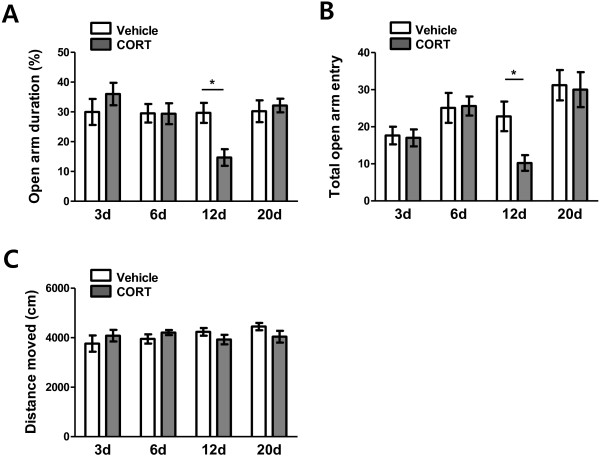
**Temporal change in anxiety-like behavior in the EPM following acute CORT treatment.** The increased anxiety level 12 days after acute CORT treatment was evident by a decrease in open arm duration **(A)** and a decrease in total number of open arm entry **(B)**. *, P < 0.05 (*post hoc* Bonferroni test). No difference in total moving distance between vehicle- and CORT-treated groups indicates that the higher anxiety-level was not accompanied by difference in locomotion activity **(C)**.

We analyzed the differences in detail between vehicle-treated and CORT-treated groups at each time point by *post-hoc* Bonferroni test following a two-way ANOVA. On day 3, no significant difference between vehicle- and CORT-treated groups was found either in total dendritic length (vehicle: 1028.2 ± 47.1 μm, CORT: 870.8 ± 48.9 μm, P > 0.05, Figures 2A-3d) or in number of branch points (vehicle: 10.52 ± 0.54, CORT: 9.38 ± 0.59, P > 0.05, Figures 2B-3d). To investigate the effects of acute CORT treatment on dendritic architecture in greater detail, segmental analysis was performed to track changes in dendritic length as a function of radial distance from the soma (segmental distance: 20 μm; Figure 2C). This analysis further confirmed that all the aspects of dendritic morphology were comparable between vehicle- and CORT-treated groups on day 3 (Figures 2C-3d).

On day 6, CORT-treated group showed slightly increased total dendritic length compared to vehicle-treated group, although the difference was not statistically significant (16.3% increase; vehicle: 972.9 ± 38.3 μm, CORT: 1131.9 ± 55.5 μm; P > 0.05; Figure 2A-6d). Number of branch points of CORT-treated group was comparable to that of vehicle-treated group (vehicle: 11.19 ± 0.46, CORT: 11.31 ± 0.56, P > 0.05, Figure 2B-6d). However, segmental analysis revealed significant dendritic expansion at 80 μm (P < 0.01) and 100 μm (P < 0.05) from the soma in CORT-treated group (Figure 2C-6d). Thus, hypertrophic effect of acute CORT began appearing 6 days after the treatment.

On day 12, CORT treatment dramatically increased total apical dendritic length (77.7% increase; vehicle: 1079.9 ± 76.2 μm, CORT: 1918.9 ± 55.2 μm, P < 0.0001, Figure 2A-12d) and total number of branch points (35.7% increase; vehicle: 12.17 ± 0.58, CORT: 16.52 ± 0.81, P < 0.0001, Figure 2B-12d). Segmental analysis also showed dendritic hypertrophy in CORT-treated group at 40 μm (P < 0.01) and within a distance of 60 ~ 160 μm (P < 0.001) from the soma (Figure 2C-12d).

On day 20, interestingly, all the dendritic measurements revealed that the dramatic hypertrophic effect was returned to the level of vehicle-treated groups, which indicates the hypertrophic effect of CORT on dendrites of BLA neurons was reversible. Total dendritic length (vehicle: 1060.9 ± 63.2 μm; CORT: 1001.6 ± 45.4 μm; P > 0.5; Figure 2A-20d) and number of branch points (vehicle: 10.71 ± 0.99; CORT: 10.10 ± 0.52; p > 0.5; Figure 2B-20d) returned to the vehicle-treated level, and any significant changes in detailed dendritic architecture were not observed (Figure 2C-20d).

Representative camera lucida drawings of Golgi-impregnated BLA pyramidal neurons for vehicle- or CORT- treated groups with various delay periods after the treatment are illustrated in Figure 2D.

### Anxiety

Stressful events induce glucocorticoid release and evoke anxiety. Amygdalar hyperactivity is known to be accompanied by anxiety-like behavior [17-20]. Therefore, we examined whether acute treatment with single dose of CORT leads to a temporal change in anxiety behavior which is paralleled by the delayed yet reversible temporal change in dendritic architecture of BLA. Anxieties of animals 3, 6, 12 and 20 days following an acute vehicle or CORT treatment were measured in terms of reduction in open arm exploration in the elevated plus maze (Figure 3). Ten to eleven animals were assigned to each condition, i.e., total 84 animals were used for the anxiety measurement.

As shown in Figure 3A, a two-way ANOVA revealed significant main effects of time (F_(3, 76)_ = 3.89, P < 0.05) and of interaction (F_(3, 76)_ = 3.17, P < 0.05) on open arm duration. However, main effect of treatment did not reach statistical significance (F_(1, 76)_ = 0.53, P > 0.5). In terms of open arm entry, only main effect of time was significant (F_(3, 76)_ = 6.76, P = 0.0004, Figure 3B). *Post hoc* Bonferroni test showed that percent open arm duration (P > 0.05, Figure 3A-3d, -6d) and number of total open arm entry (P > 0.05, Figure 3B-3d, -6d) were comparable between vehicle- and CORT-treated groups 3 days and 6 days after the treatments. Meanwhile, on day 12, CORT-treated group exhibited a significantly greater degree of anxiety compared with vehicle-treated group. This elevated anxiety level was manifested as a significant reduction in percentage of open arm duration (48.9% reduction; vehicle: 29.16 ± 3.08%, CORT: 14.89 ± 2.50%; P < 0.05; Figure 3A-12d) and number of open arm entry (55.2% reduction; vehicle: 23.90 ± 3.80, CORT: 10.70 ± 1.95; P < 0.05; Figure 3B-12d).

Interestingly, the enhanced anxiety was not persistent. On day 20, anxiety level of CORT-treated group returned to that of vehicle-treated group; both open arm duration (vehicle: 30.76 ± 3.35%, CORT: 32.15 ± 2.30%; P > 0.05; Figure 3A-20d) and open arm entry (vehicle: 30.36 ± 3.80, CORT: 30.00 ± 4.71; P > 0.05; Figure 3B-20d) of CORT-treated group were comparable to those of vehicle-treated group.

As shown in Figure 3C, a two-way ANOVA revealed no significant main effects of interaction (F_(3, 76)_ = 1.62, P > 0.1), of treatment (F_(1, 76)_ = 0.057, P < 0.8) and of time (F_(3, 76)_ = 0.79, P > 0.5) on total moving distance, which implicates that the higher anxiety was not accompanied by difference in locomotion activity. Overall, acute CORT induced dendritic hypertrophy of BLA spiny neurons, which was paralleled by heightened anxiety, both peaked 12 days after the treatment. Therefore, dendritic arborization of BLA neurons was closely related with anxiety level.

### Dendritic architecture of mPFC neurons

**Figure 4:**
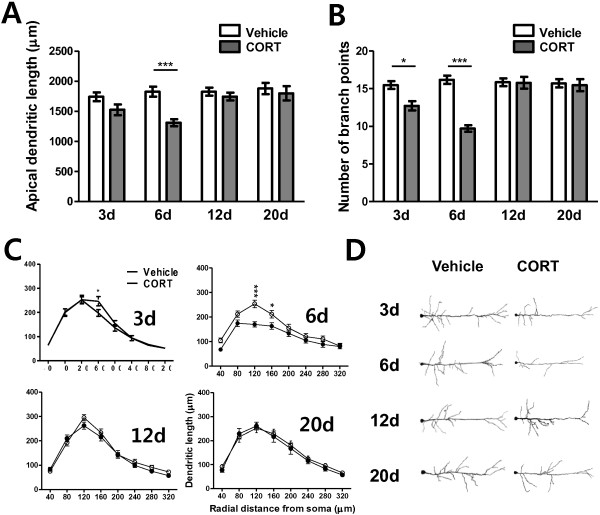
**Temporal changes in dendritic arborization of mPFC neurons following acute CORT treatment.** Acute CORT treatment elicited a decrease in dendritic arborization 3 and 6 days after the treatment. The decrease in dendritic arborization was manifested by decreased total dendritic length **(A)** and total number of branch points **(B)**, and segmental analysis revealed an increase in dendritic arborization in the segments between 120 μm and 160 μm from the cell body **(C)**. Representative camera lucida drawings of Golgi-impregnated mPFC pyramidal neurons for vehicle- or CORT- treated groups with various delay periods after the treatment **(D)**. Only apical dendrites were depicted for clarity. *, P < 0.05; ***, P < 0.001 (*post hoc* Bonferroni test).

In addition to the BLA, the mPFC has been known to be one of the stress vulnerable brain areas. The mPFC in rodents includes the dorsal anterior cingulate cortex (ACd), prelimbic PFC (PL) and infralimbic PFC (IL). Apical dendritic arborization of pyramidal neurons located in the PL and IL was measured across days in terms of total dendritic length (Figure 4A) and number of branch points (Figure 4B). A detailed segmental analysis was also performed using radial distance from the soma (Segmental distance, 40 μm; Figure 4C). Seven animals were assigned to each condition and 20 ~ 27 neurons (about 3 neurons per an animal) were used for each measurement.

Overall, delayed and reversible atrophic alteration of dendritic arborization of mPFC neurons upon acute CORT treatment was observed. In terms of total apical dendritic length, a two-way ANOVA revealed significant main effects of time (F_(3,48_ = 4.63, P = 0.0064), of treatment (F_(1, 48)_ = 14.44, P = 0.0004), and of their interaction (F_(3, 48)_ = 3.03, P = 0.0381). Number of branch points of mPFC neurons also showed significant main effects of time (F_(3, 48)_ = 9.77, P < 0.0001), of treatment (F_(1, 48)_ = 30.57, P < 0.0001) and of their interaction (F_(3, 48)_ = 11.96, P < 0.0001). The difference between vehicle-treated and CORT-treated groups at each time point was further analyzed in detail by *post-hoc* Bonferroni test following a two-way ANOVA.

On day 3, there was a trend of decrease in dendritic arborization in the CORT-treated group compared with the vehicle-treated group. Although a decrease of total dendritic length in the CORT-treated group did not reach statistical significance (12.4% decrease; vehicle: 1739.9 ± 74.3 μm, CORT: 1523.6 ± 89.7 μm, P > 0.05, Figure 4A-3d), number of branch points significantly decreased in the CORT-treated group (15.8% decrease; vehicle: 15.48 ± 0.52, CORT: 12.72 ± 0.63, P < 0.05, Figure 4B-3d). Segmental analysis (segmental distance: 40 μm) also revealed that dendritic atrophy in the CORT-treated group began to be apparent at 160 μm from soma on day 3 (P < 0.05, Figure 4C-3d).

On day 6, CORT-treated group showed a significant decrease in total dendritic length (28.2% decrease; vehicle: 1824.9 ± 82.7 μm, CORT: 1309.8 ± 57.7 μm, P < 0.001, Figure 4A-6d) as well as in number of branch points (39.9% decrease; vehicle: 16.18 ± 0.55, CORT: 9.71 ± 0.44, P < 0.001, Figure 4B-6d). Segmental analysis revealed significant dendritic atrophy at 120 μm (P < 0.001) and 160 μm (P < 0.05) from soma in CORT-treated group (Figure 4C-6d).

However, 12 days after an acute CORT treatment, atrophic effects of CORT on dendrite morphology dramatically disappeared, which maintained thereafter; total dendritic length (vehicle: 1823.8 ± 66.8 μm, CORT: 1743.4 ± 65.3 μm, P > 0.05, Figure 4A-12d) and number of branch points (vehicle: 15.86 ± 0.49, CORT: 15.77 ± 0.80, P > 0.05, Figure 4B-12d) returned to the vehicle-treated level and no significant changes in detailed dendritic architecture were observed (Figure 4C-12d). Also, both total apical dendritic length (vehicle: 1877.9 ± 93.6 μm; CORT: 1796.4 ± 118.6 μm; P > 0.5; Figure 4A-20d) and total number of branch points (vehicle: 15.71 ± 0.54; CORT: 15.47 ± 0.812; p > 0.5; Figure 4B-20d) were comparable. The segmental analysis also confirmed the disappearance of CORT effect on day 20 (Figure 4C-20d). Therefore, the atrophic effect of CORT on dendrite of mPFC neurons is reversible. Figure 4D shows camera lucida drawings of representative Golgi-impregnated mPFC pyramidal neurons from vehicle- and CORT-treated animals.

### Working memory

Apical dendritic atrophy and spine loss in mPFC are structural changes that result from experiencing traumatic stress [12,16,28,29] and these changes may be associated with altered emotionality, impaired working memory, and dysfunctional regulation of stress hormone homeostasis [11,30-33]. In the present study, we found that alteration of the anxiety level after an acute CORT treatment follows the time course change of dendritic architecture of BLA neurons but not mPFC neurons, indicating that enhanced anxiety is more likely associated with BLA hypertrophy rather than mPFC atrophy. Therefore, we investigated whether mPFC atrophy upon acute CORT treatment is rather accompanied by working memory deficit.

**Figure 5:**
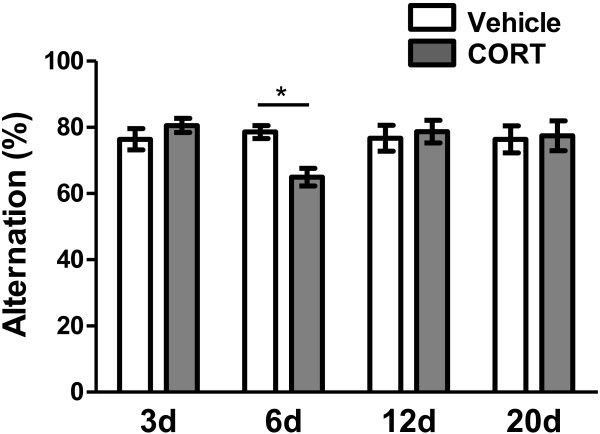
**Temporal change in performance of working memory following acute CORT treatment.** Impaired working memrory occurred 6 days after acute CORT treatment as evident by a decrease in percent of spontaneous alternations. *, P < 0.05 (*post hoc* Bonferroni test).

We assessed the temporal effect of acute CORT treatment on working memory performance using the Y-maze test. Percentage alternation calculated as the ratio of actual to possible alternation was considered as a parameter for working memory-related behaviors. Percent alternations of animals in the Y-maze 3, 6, 12 and 20 days following an acute vehicle or CORT treatment were measured (Figure 5). Ten animals were assigned to each condition and total 80 animals were used for the anxiety measurement. As shown in Figure 5, a two-way ANOVA revealed a significant main effect of interaction (F_(3, 72)_ = 2.94, P < 0.05). However, main effect of treatment (F_(1, 72)_ = 0.47, P > 0.5) and of time (F_(3, 72)_ = 1.64, P > 0.1) did not reach statistical significance. *Post hoc* Bonferroni test showed that working memory impairment only in the CORT-treated animals on day 6 was manifested as a significant reduction in percentage of spontaneous alternations (17.3% reduction; vehicle: 78.59 ± 1.93%, CORT: 64.95 ± 2.68%; P < 0.05; Figure 5-6d).

Regardless of CORT treatment, however, percentage of alternation was comparable to the vehicle-treated animals on days 3, 12 and 20 (P > 0.05, Figure 5-3d, -12d, -20d). Our result clearly shows coincident occurrence of the working memory impairment and the dendrite atrophy of mPFC neurons in CORT-treated group 6 day after the treatment. There was a strong trend that high and low level of dendritic arborizations of mPFC neurons is accompanied by high and low performance of working memory, respectively.

## Discussion

We first examined the time-course effect of an acute CORT administration on dendritic architectures in the BLA and investigated the relationship between the dendritic remodeling in the BLA and anxiety across time. We found that change in dendritic arborization of BLA neurons is paralleled by alteration in anxiety; peaked dendritic hypertrophy of BLA neurons was concomitant with culminating anxiety behavior at 12d following acute CORT treatment (Figures 2, 3). Behavioral alterations found in stress-related mental illness are considered to be closely related with morphological changes of neurons in a certain brain regions. Indeed, growing body of evidence indicates that anxiety generated by stress and stress hormone is caused by dendritic hypertrophy in BLA neurons. For instance, chronic immobilization stress increases dendritic length and number of branch points of BLA neurons [18] and this dendritic remodeling is accompanied by enhanced anxiety [19,34]. More importantly, experimental reduction of dendritic length results in reduced anxiety [35]. Therefore, our result is consistent with the observations that BLA hypertrophy is relevant to the heightened anxiety upon stress.

While chronic immobilization stress successfully produces BLA hypertrophy and enhanced anxiety, chronic unpredictable stress fails to show both dendritic elongation [18] and anxiety-like behavior [19]. Single acute immobilization stress causes increased spine density and heightened anxiety, without changing dendritic arborization [17]. Our present study further confirms that acute treatment with a single dose of glucocorticoid elicits neuronal hypertrophy in the BLA and enhanced anxiety 12 days after the treatment [20]. However, previous study reports that effect of repeated CORT administration on dendritic morphology appears to be brain region-specific and chronic CORT administration for 21 days shows no morphological changes in BLA pyramidal neurons [36]. Overall, these findings suggest that certain forms of stress may affect BLA hypertrophy and thereby lead to enhanced anxiety. In addition, length of stress exposure time may affect BLA differently.

Neurons in the mPFC are also highly sensitive to stress. However, in contrast to hypertrophic effect on BLA neurons, stress decreases dendritic branching and length of pyramidal neurons in the mPFC [12]. Chronic stress can induce dendritic spine loss in mPFC pyramidal neurons [37]. These dendritic remodeling can be induced by repeated chronic stress or by acute intense stress [32,38-40]. Glucocorticoid receptors is enriched in the mPFC [15] and activation of glucocorticoid receptors with chronic treatment of CORT mimics the effects of chronic stress on the mPFC [16,32,41]. Now, we asked whether acute treatment with a single dose of CORT triggers dendritic remodeling in mPFC pyramidal neurons. As expected, acute CORT treatment also produced changes in dendritic arborization of mPFC neurons as found in BLA neurons yet with distinct characteristics (Figure 4). CORT produced apical dendritic atrophy in mPFC neurons rather than hypertrophy. Moreover, the structural change in mPFC pyramidal neurons was also reversible but it was less delayed compared with that in BLA neurons. Dendritic atrophy of mPFC neurons was peaked 6 days after acute CORT treatment, which is about a week earlier than the CORT-induced dendritic hypertrophy of BLA neurons. Remarkably, dendritic atrophy in mPFC neurons is not paralleled by anxiety behavior. This finding seems to be inconsistent with the accumulating data suggesting that the mPFC in addition to the BLA is preferentially involved in anxiety.

An increasing number of studies demonstrate that the mPFC is implicated in emotional processing in both physiological and pathological states. In humans, decreased ventral mPFC volume, lower activity than average and reduced connectivity to limbic systems are associated with the prevalence of anxiety and panic disorders [42], major depression [42,43], phobias [44], and PTSD [45]. Similar to humans, the rodent mPFC also regulates anxiety behaviors. Rats bred for high anxiety also show lower baseline activity and reduced tissue oxygenation in mPFC [46]. Furthermore, animals exhibiting higher basal anxiety-like behavior have shorter apical dendrites in layer II/III pyramidal neurons of the mPFC compared to their low anxiety counterparts [47]. Chronic stress that has been shown to increase anxiety in rodents also generates dendritic remodeling of mPFC pyramidal neurons [12].

A clue to explain the discrepancy between our present observation and others seems to lie on the previous finding that effect of stress on dendritic remodeling is circuit-specific; a subpopulation of mPFC neurons that project to the BLA is resistant to stress-induced dendritic atrophy [48]. Thus, if a small subpopulation of neurons that participate in anxiety behavior is resistant to stress-related dendritic remodeling, they will function normally regardless of pre-exposure to stress, thereby maintaining unaltered anxiety level.

A number of behavioral studies demonstrate that stress-induced impaired performance of working memory test specifically depends on the integrity of the PFC [31,32]. The decreased dendritic arborization in the mPFC in chronically stressed rats predicts impairment of attentional set-shifting performance [31]. Therefore, we tested the possibility that stress-vulnerable mPFC neurons exhibiting dendritic atrophy participate in working memory performance. Our study shows that occurrence of working memory impairment coincided with the dendritic atrophy of mPFC neurons. Therefore, impaired working memory rather than enhanced anxiety might be a behavioral consequence of the stress-induced decrease in dendritic arborization in mPFC pyramidal neurons. Nevertheless, we should note that the present evidence for behavioral consequence of stress-induced dendritic changes in the amygdala and mPFC is rather correlational in nature. Therefore, it is crucial to understand circuit-specificity of stress-induced dendritic remodeling as pursued in the previous study [48].

A remarkable finding in our study is that both robust hypertrophy of the BLA and enhanced anxiety are readily reversible. While dendritic remodeling of mPFC neurons caused by stress has been known to be reversible [49], dendritic hypertrophy in BLA neurons as well as enhanced anxiety, once generated, long-lasts and fails to reverse with recovery [34]. However, the present study revealed for the first time that dendritic hypertrophy of BLA neurons and its associated heightened anxiety can be fully recovered.

In PTSD, a single traumatic event triggers changes in behaviors including anxiety that are both delayed and prolonged [21,22], which accompanied by BLA hyperactivity [23]. Exposure to severe stress like a life-threatening event is the precipitating factor for PTSD. However, not every individual experiencing traumatic stress develops the stress-related mental illness. Upon exposure to traumatic stress, PTSD-like symptoms are apparent in almost all people in the days and weeks but the symptoms gradually disappear thereafter in the majority [27]. Thus, the loss of resilience may have relevance to PTSD. Because stress-induced changes of dendritic morphology and related behavioral outcomes appear to be readily reversible in our stress model, further studies on cellular and molecular mechanisms of recovery-related plasticity using this model might provide a new insight into the etiological factors of stress-related mental illness such as PTSD.

## Conclusions

Our results further confirm that heightened anxiety is tightly correlated with hypertrophic effect of stress on BLA neurons. Working memory deficit appears to be functional consequence of morphological changes in mPFC neurons. Therefore, the stress-induced structural changes in the BLA and the mPFC might be associated specifically with distinct behavioral symptoms observed in stress-related mental illness. Because the stress-induced changes of dendritic morphology and related behavioral outcomes appear to be readily reversible, further studies on cellular mechanism of plastic changes of dendrites might provide new insight into the etiological factors of stress-related mental illness such as PTSD.

## Methods

### Experimental animals

Male Sprague–Dawley rats (6 weeks of age at the beginning of experiments) were used in all experiments. All rats were housed 2 ~ 3 animals per a cage in a temperature-controlled room on a 12 h light/dark cycle with the light period beginning at 7:00 P.M. Food and water were available ad libitum. The experimental groups were divided into 8 groups (3d-vehicle, 3d-CORT, 6d-vehicle, 6d-CORT, 12d-vehicle, 12d-CORT, 20d-vehicle and 20d-CORT groups) and rats were randomly assigned to each group. The rest of the groups were examined accordingly with the experimental schedule as shown in Figure 1A. Acute CORT treatment consisted of a single s.c. injection of CORT (10 mg/kg of body weight) dissolved in 60% ethanol (in 0.9% saline). Rats treated with 60% ethanol alone were served as controls (vehicle-treated group). All animal procedures were approved (KHUASP(SE)-13-031) and monitored by the Kyung Hee University Institutional Animal Care and Use Committee.

### Morphological studies

Rats were decapitated under deep anesthesia with isoflurane. Brain tissue was prepared by using the rapid Golgi kit (FD Neurotechnologies) according to the manufacturer’s instructions. Following a 14 day incubation period, brain tissue was sliced coronally (150 μm) using a HA752 vibrotome (Campden) and mounted on gelatin-coated slides. Sections were collected serially, dehydrated in absolute alcohol, cleared in xylene, and coverslipped. Golgi-impregnated spiny neurons in the BLA (-2.3 to -3.6 relative to bregma) and pyramidal neurons lying in layer II/III of the mPFC (+3.3 to +2.8 relative to bregma) were studied according to the atlas of Paxinos and Watson [50]. Images of selected Golgi-impregnated neuron were captured with Axiocam HRC (ZEISS) in a Axioskop 50 (ZEISS) using Plan-Neofluor objective 100X/1.40 oil, saved as 16-bit TIFF files using AxioVision program, (ZEISS). Camera lucida drawing was generated by tracing a neuron of the overlay image in a new layer with “line tool” in Photoshop program (Adobe Systems) as previously described [51]. Neurons with a minimal overlap of dendrites, heavily impregnated with silver nitrate and without truncated dendrites were selected for drawing. Morphometric analysis (calculation of total dendritic length and total number of branch points) of digitized images was performed by using ImageJ program (NIH).

### The Elevated Plus Maze (EPM)

The behavioral tests were carried out in a behavior-test room (temperature approximately 24 °C, light level 40 lux). All tests were carried out between the 10:00–17:00 h (dark phase of daily cycle). The EPM test is based on the natural aversion of rodent for open and elevated areas, as well as on their natural spontaneous exploratory behavior in novel environments. This test is the best characterized and most frequently used animal model for measuring anxiety [52]. The EPM consisted of two opposite open arms and two opposite closed arms. Two open arms were 50 cm × 10 cm and two closed arms were 50 cm × 10 cm and enclosed by 30 cm high walls on each side and ends. The closed arms were made of black acryl. The center platform was 10 cm × 10 cm. The maze was elevated 50 cm from the floor. At the beginning of each trial, a rat was placed into the center of the plus maze facing an open arm. Each trial lasted 10 min. The elevated plus maze was cleaned with 70% ethanol solution after each trial. The number of open arm entries and time spent (duration) in open arms were measured as open-arm exploration. A rat was considered to have entered an arm when all four paws were positioned within an arm. In this paradigm, hightened anxiety is defined as a decreased open-arm exploration. EthoVision program (Noldus Information Tech.) was used for data analysis.

### The Y-maze

The Y-maze spontaneous alternation paradigm is based on the natural tendency of rodents to explore a novel environment. When placed in the Y-maze, mice will explore the least recently visited arm, and thus tend to alternate visits between the three arms. For efficient alternation, mice need to use working memory, and thus, they should maintain an ongoing record of most recently visited arms, and continuously update such a record [53]. The Y-maze consisted of three arms that were 50 cm long, 15 cm wide and 20 cm high. Three arms were converged at an equilateral triangle center, placed 120 degree with respect to each other. The maze was made of black-acryl. Three arms had visual cues that consisted of a star, a triangle and a circle form. Rats were placed into the end of the start arm and allowed to freely explore the maze for 8 min. Arm entry session were recorded when a rat’s hind legs were placed into two thirds of the arm. Spontaneous alternation was defined as successive entries into the three arms on overlapping triplet sets. The percentage alternation was calculated as the ratio of actual to possible alternation (defined as the total number of arm entries minus two) × 100 and considered as a parameter for working memory-related behaviors.

### Statistical analysis

All sampling of morphological and behavioral studies occurred with an investigator blind to groups. Statistical differences were determined by two-way analysis of variance (ANOVA) with acute CORT treatment (CORT or vehicle) and time after the treatment (3d, 6d, 12d, and 20d) as between-group sources of variance. For segmental analysis (e.g., for a within-group source of variance), statistical differences were determined by repeated measures two-way ANOVA. *Post hoc* Bonferroni analysis was performed for multiple comparisons with P < 0.05 considered significant (Graphpad Prism software 5.0). The F values and experimental degrees of freedom are included in Results. Data are presented as mean ± SEM, and percentage change are calculated with respect to corresponding control values.

## Abbreviations

CORT: Corticosterone; BLA: Basolateral amygdala; mPFC: Medial prefrontal cortex; PTSD: Posttraumatic stress disorder; PL: Prelimbic cortex; IL: Infralimbic cortex; EPM: Elevated plus maze.

## Competing interests

The authors declare that there are no competing interests.

## Authors’ contributions

HK carried out the morphological studies, participated in the behavioural experiments and drafted the manuscript. JHY and KC carried out he behavioural experiments. SH, SJK and KSS analysed data. SJK and KSS conceived of the study, and participated in its design and coordination and wrote the paper. All authors read and approved the final manuscript.

## References

[B1] Tasker JG Herman JP Mechanisms of rapid glucocorticoid feedback inhibition of the hypothalamic-pituitary-adrenal axis Stress 2011 15 398 406 21663538 10.3109/10253890.2011.586446 PMC4675656

[B2] Smith SM Vale WW The role of the hypothalamic-pituitary-adrenal axis in neuroendocrine responses to stress Dialogues Clin Neurosci 2006 15 383 395 17290797 10.31887/DCNS.2006.8.4/ssmith PMC3181830

[B3] McEwen BS Stress and hippocampal plasticity Annu Rev Neurosci 1999 15 105 122 10.1146/annurev.neuro.22.1.105 10202533

[B4] Magarinos AM McEwen BS Stress-induced atrophy of apical dendrites of hippocampal CA3c neurons: involvement of glucocorticoid secretion and excitatory amino acid receptors Neuroscience 1995 15 89 98 10.1016/0306-4522(95)00259-L 8637636

[B5] Chiba S Numakawa T Ninomiya M Richards MC Wakabayashi C Kunugi H Chronic restraint stress causes anxiety- and depression-like behaviors, downregulates glucocorticoid receptor expression, and attenuates glutamate release induced by brain-derived neurotrophic factor in the prefrontal cortex Prog Neuropsychopharmacol Biol Psychiatry 2012 15 112 119 10.1016/j.pnpbp.2012.05.018 22664354

[B6] Akirav I Cannabinoids and glucocorticoids modulate emotional memory after stress Neurosci Biobehav Rev 2013 15 2554 2563 10.1016/j.neubiorev.2013.08.002 23954749

[B7] Timmermans W Xiong H Hoogenraad CC Krugers HJ Stress and excitatory synapses: from health to disease Neuroscience 2013 15 626 636 23727506 10.1016/j.neuroscience.2013.05.043

[B8] de Quervain DJ Aerni A Schelling G Roozendaal B Glucocorticoids and the regulation of memory in health and disease Front Neuroendocrinol 2009 15 358 370 10.1016/j.yfrne.2009.03.002 19341764

[B9] Bush G Luu P Posner MI Cognitive and emotional influences in anterior cingulate cortex Trends Cogn Sci 2000 15 215 222 10.1016/S1364-6613(00)01483-2 10827444

[B10] Kern S Oakes TR Stone CK McAuliff EM Kirschbaum C Davidson RJ Glucose metabolic changes in the prefrontal cortex are associated with HPA axis response to a psychosocial stressor Psychoneuroendocrinology 2008 15 517 529 10.1016/j.psyneuen.2008.01.010 18337016 PMC2601562

[B11] Moghaddam B Jackson M Effect of stress on prefrontal cortex function Neurotox Res 2004 15 73 78 10.1007/BF03033299 15184108

[B12] Cook SC Wellman CL Chronic stress alters dendritic morphology in rat medial prefrontal cortex J Neurobiol 2004 15 236 248 10.1002/neu.20025 15266654

[B13] Rauch SL Shin LM Segal E Pitman RK Carson MA McMullin K Whalen PJ Makris N Selectively reduced regional cortical volumes in post-traumatic stress disorder Neuroreport 2003 15 913 916 12802174 10.1097/01.wnr.0000071767.24455.10

[B14] Yamasue H Kasai K Iwanami A Ohtani T Yamada H Abe O Kuroki N Fukuda R Tochigi M Furukawa S Sadamatsu M Sasaki T Aoki S Ohtomo K Asukai N Kato N Voxel-based analysis of MRI reveals anterior cingulate gray-matter volume reduction in posttraumatic stress disorder due to terrorism Proc Natl Acad Sci U S A 2003 15 9039 9043 10.1073/pnas.1530467100 12853571 PMC166434

[B15] Diorio D Viau V Meaney MJ The role of the medial prefrontal cortex (cingulate gyrus) in the regulation of hypothalamic-pituitary-adrenal responses to stress J Neurosci 1993 15 3839 3847 8396170 10.1523/JNEUROSCI.13-09-03839.1993 PMC6576467

[B16] Wellman CL Dendritic reorganization in pyramidal neurons in medial prefrontal cortex after chronic corticosterone administration J Neurobiol 2001 15 245 253 10.1002/neu.1079 11745662

[B17] Mitra R Jadhav S McEwen BS Vyas A Chattarji S Stress duration modulates the spatiotemporal patterns of spine formation in the basolateral amygdala Proc Natl Acad Sci U S A 2005 15 9371 9376 10.1073/pnas.0504011102 15967994 PMC1166638

[B18] Vyas A Mitra R Shankaranarayana RBS Chattarji S Chronic stress induces contrasting patterns of dendritic remodeling in hippocampal and amygdaloid neurons J Neurosci 2002 15 6810 6818 12151561 10.1523/JNEUROSCI.22-15-06810.2002 PMC6758130

[B19] Vyas A Chattarji S Modulation of different states of anxiety-like behavior by chronic stress Behav Neurosci 2004 15 1450 1454 15598155 10.1037/0735-7044.118.6.1450

[B20] Mitra R Sapolsky RM Acute corticosterone treatment is sufficient to induce anxiety and amygdaloid dendritic hypertrophy Proc Natl Acad Sci U S A 2008 15 5573 5578 10.1073/pnas.0705615105 18391224 PMC2291109

[B21] American Psychiatric Association Diagnostic and Statistical Mental Disorders: DSM-IV-TR 2000 Washington, DC: American Psychiatric Publishing

[B22] Yehuda R Post-traumatic stress disorder N Engl J Med 2002 15 108 114 10.1056/NEJMra012941 11784878

[B23] Yehuda R LeDoux J Response variation following trauma: a translational neuroscience approach to understanding PTSD Neuron 2007 15 1 19 32 10.1016/j.neuron.2007.09.006 17920012

[B24] Kessler RC Sonnega A Bromet E Hughes M Nelson CB Posttraumatic stress disorder in the National Comorbidity Survey Arch Gen Psychiatry 1995 15 1048 1060 10.1001/archpsyc.1995.03950240066012 7492257

[B25] Breslau N Reboussin BA Anthony JC Storr CL The structure of posttraumatic stress disorder: latent class analysis in 2 community samples Arch Gen Psychiatry 2005 15 1343 1351 10.1001/archpsyc.62.12.1343 16330722

[B26] Breslau N Peterson EL Poisson LM Schultz LR Lucia VC Estimating post-traumatic stress disorder in the community: lifetime perspective and the impact of typical traumatic events Psychol Med 2004 15 889 898 10.1017/S0033291703001612 15500309

[B27] McFarlane AC Posttraumatic stress disorder: a model of the longitudinal course and the role of risk factors J Clin Psychiatry 2000 15 Suppl 5 15 20 discussion 21–13 10761675

[B28] Radley JJ Sisti HM Hao J Rocher AB McCall T Hof PR McEwen BS Morrison JH Chronic behavioral stress induces apical dendritic reorganization in pyramidal neurons of the medial prefrontal cortex Neuroscience 2004 15 1 6 10.1016/j.neuroscience.2004.01.006 15051139

[B29] Radley JJ Rocher AB Miller M Janssen WG Liston C Hof PR McEwen BS Morrison JH Repeated stress induces dendritic spine loss in the rat medial prefrontal cortex Cereb Cortex 2006 15 313 320 15901656 10.1093/cercor/bhi104

[B30] Quirk GJ Beer JS Prefrontal involvement in the regulation of emotion: convergence of rat and human studies Curr Opin Neurobiol 2006 15 723 727 10.1016/j.conb.2006.07.004 17084617

[B31] Liston C Miller MM Goldwater DS Radley JJ Rocher AB Hof PR Morrison JH McEwen BS Stress-induced alterations in prefrontal cortical dendritic morphology predict selective impairments in perceptual attentional set-shifting J Neurosci 2006 15 7870 7874 10.1523/JNEUROSCI.1184-06.2006 16870732 PMC6674229

[B32] Cerqueira JJ Pego JM Taipa R Bessa JM Almeida OF Sousa N Morphological correlates of corticosteroid-induced changes in prefrontal cortex-dependent behaviors J Neurosci 2005 15 7792 7800 10.1523/JNEUROSCI.1598-05.2005 16120780 PMC6725252

[B33] Sullivan RM Gratton A Prefrontal cortical regulation of hypothalamic-pituitary-adrenal function in the rat and implications for psychopathology: side matters Psychoneuroendocrinology 2002 15 99 114 10.1016/S0306-4530(01)00038-5 11750772

[B34] Vyas A Pillai AG Chattarji S Recovery after chronic stress fails to reverse amygdaloid neuronal hypertrophy and enhanced anxiety-like behavior Neuroscience 2004 15 667 673 10.1016/j.neuroscience.2004.07.013 15464275

[B35] Mitra R Ferguson D Sapolsky RM SK2 potassium channel overexpression in basolateral amygdala reduces anxiety, stress-induced corticosterone secretion and dendritic arborization Mol Psychiatry 2009 15 847 855 827 10.1038/mp.2009.9 19204724 PMC2763614

[B36] Morales-Medina JC Sanchez F Flores G Dumont Y Quirion R ReMorphological reorganization after repeated corticosterone administration in the hippocampus, nucleus accumbens and amygdala in the rat J Chem Neuroanat 2009 15 266 272 10.1016/j.jchemneu.2009.05.009 19505571

[B37] Radley JJ Rocher AB Rodriguez A Ehlenberger DB Dammann M McEwen BS Morrison JH Wearne SL Hof PR Repeated stress alters dendritic spine morphology in the rat medial prefrontal cortex J Comp Neurol 2008 15 1141 1150 10.1002/cne.21588 18157834 PMC2796421

[B38] Seib LM Wellman CL Daily injections alter spine density in rat medial prefrontal cortex Neurosci Lett 2003 15 29 32 10.1016/S0304-3940(02)01287-9 12524164

[B39] Brown SM Henning S Wellman CL Mild, short-term stress alters dendritic morphology in rat medial prefrontal cortex Cereb Cortex 2005 15 1714 1722 10.1093/cercor/bhi048 15703248

[B40] Izquierdo A Wellman CL Holmes A Brief uncontrollable stress causes dendritic retraction in infralimbic cortex and resistance to fear extinction in mice J Neurosci 2006 15 5733 5738 10.1523/JNEUROSCI.0474-06.2006 16723530 PMC6675270

[B41] Cerqueira JJ Taipa R Uylings HB Almeida OF Sousa N Specific configuration of dendritic degeneration in pyramidal neurons of the medial prefrontal cortex induced by differing corticosteroid regimens Cereb Cortex 2007 15 1998 2006 10.1093/cercor/bhl108 17082516

[B42] van Tol MJ van der Wee NJ van den Heuvel OA Nielen MM Demenescu LR Aleman A Renken R van Buchem MA Zitman FG Veltman DJ Regional brain volume in depression and anxiety disorders Arch Gen Psychiatry 2010 15 1002 1011 10.1001/archgenpsychiatry.2010.121 20921116

[B43] Savitz JB Drevets WC Imaging phenotypes of major depressive disorder: genetic correlates Neuroscience 2009 15 300 330 10.1016/j.neuroscience.2009.03.082 19358877 PMC2760612

[B44] Hermann A Schafer A Walter B Stark R Vaitl D Schienle A Emotion regulation in spider phobia: role of the medial prefrontal cortex Soc Cogn Affect Neurosci 2009 15 257 267 10.1093/scan/nsp013 19398537 PMC2728632

[B45] Etkin A Wager TD Functional neuroimaging of anxiety: a meta-analysis of emotional processing in PTSD, social anxiety disorder, and specific phobia Am J Psychiatr 2007 15 1476 1488 10.1176/appi.ajp.2007.07030504 17898336 PMC3318959

[B46] Kalisch R Salome N Platzer S Wigger A Czisch M Sommer W Singewald N Heilig M Berthele A Holsboer F Landqraf R Auer DP High trait anxiety and hyporeactivity to stress of the dorsomedial prefrontal cortex: a combined phMRI and Fos study in rats Neuroimage 2004 15 382 391 10.1016/j.neuroimage.2004.06.012 15325386

[B47] Miller MM Morrison JH McEwen BS Basal anxiety-like behavior predicts differences in dendritic morphology in the medial prefrontal cortex in two strains of rats Behav Brain Res 2012 15 280 288 10.1016/j.bbr.2012.01.029 22285422

[B48] Shansky RM Hamo C Hof PR McEwen BS Morrison JH Stress-induced dendritic remodeling in the prefrontal cortex is circuit specific Cereb Cortex 2009 15 2479 2484 10.1093/cercor/bhp003 19193712 PMC2742599

[B49] Radley JJ Rocher AB Janssen WG Hof PR McEwen BS Morrison JH Reversibility of apical dendritic retraction in the rat medial prefrontal cortex following repeated stress Exp Neurol 2005 15 199 203 10.1016/j.expneurol.2005.07.008 16095592

[B50] Paxinos R Watson C The Rat Brain in Stereotaxic Coordinates 2005 5 San Diego: Elsevier

[B51] Chung HJ Jan YN Jan LY Polarized axonal surface expression of neuronal KCNQ channels is mediated by multiple signals in the KCNQ2 and KCNQ3 C-terminal domains Proc Natl Acad Sci U S A 2006 15 8870 8875 10.1073/pnas.0603376103 16735477 PMC1472242

[B52] Pellow S Chopin P File SE Briley M Validation of open:closed arm entries in an elevated plus-maze as a measure of anxiety in the rat J Neurosci Methods 1985 15 149 167 10.1016/0165-0270(85)90031-7 2864480

[B53] Wall PM Messier C Infralimbic kappa opioid and muscarinic M1 receptor interactions in the concurrent modulation of anxiety and memory Psychopharmacology (Berl) 2002 15 233 244 10.1007/s00213-001-0979-9 11889492

